# Label-Free Optical Sensor for Real-Time Monitoring of Insulin Secretion from Single Human Pancreatic Islets

**DOI:** 10.3390/s26103069

**Published:** 2026-05-13

**Authors:** Mark F. Coughlan, Lei Zhang, Umar Khan, Xuejun Zhang, Paul K. Upputuri, Maria Glyavina, Yuri N. Zakharov, Le Qiu, Lev T. Perelman

**Affiliations:** Center for Advanced Biomedical Imaging and Photonics, Beth Israel Deaconess Medical Center, Harvard University, Boston, MA 02115, USA

**Keywords:** islet-on-a-chip, label-free, microscopy, insulin

## Abstract

Glucose-stimulated insulin secretion is the central functional readout of pancreatic islets, yet existing assays often require offline processing or pooling of multiple islets, limiting real-time assessment of single-islet function. Here we report a microscopy-compatible islet-on-a-chip (IOC) integrated with light scattering-based broadband backscattering confocal microscopy (BBCM) for continuous, label-free optical readout of insulin secretion dynamics in functional human islets. Fabricated using two-photon polymerization, the IOC-BBCM sensor stabilizes single human islets under continuous perfusion for high-resolution optical interrogation. The sensor identifies insulin-rich β-cells label-free, as confirmed by insulin immunostaining, and monitors granule depletion and redistribution during glucose and potassium chloride (KCl) stimulation, matching ELISA-quantified insulin secretion from the same perfused islets. This modular sensor provides a non-destructive, label-free approach for monitoring stimulus-linked secretion dynamics from individual human islets and should support longitudinal studies of islet function.

## 1. Introduction

Pancreatic β-cells, organized within pancreatic islets, are the primary regulators of glucose homeostasis [1]. In type 1 diabetes (T1D), these β-cells are progressively destroyed by the immune system [2], requiring lifelong insulin therapy. Cell replacement strategies, including iPSC-derived islets [3,4,5,6] and donor-derived islet transplantation [7,8], represent promising therapeutic avenues for restoring endogenous insulin production in diabetes. As these therapies advance toward broader clinical use, a major unmet need is a scalable, non-destructive sensing approach for monitoring iPSC-derived islet differentiation and maturation over time. Differentiation protocols often produce heterogeneous clusters containing polyhormonal cells and cells with limited glucose responsiveness [3,9], making one-time assays inadequate for guiding process improvements. Glucose-stimulated insulin secretion (GSIS) is the most important functional readout from iPSC-derived islets and can be observed during approximately the final 40% of the differentiation protocol [3,10]. Conditions within this window can be tuned, and perturbations to the maturation medium can substantially reshape insulin secretion dynamics over subsequent weeks [11]. Despite this, conventional workflows are not well suited for frequent measurements of small numbers of individual islets, which is problematic given that the early signs of glucose responsiveness can be obscured by measurements of pooled islets [4].

GSIS is typically measured by enzyme-linked immunosorbent assay (ELISA) [3,4]. While accurate, ELISA-based workflows are reagent-intensive, require substantial manual handling, have long processing times, and generally average secretion over large islet pools. As a result, throughput, temporal resolution, and islet-to-islet heterogeneity are difficult to assess. Microfluidic islet-on-a-chip (IOC) platforms [12] improve stimulation control and reduce sample volume, but in many designs, secretion is still quantified offline from collected effluent [13,14]. Fractionated collection improves time resolution relative to bulk assays yet remains constrained by downstream processing and kit cost. Droplet-based fractionation systems, while capable of higher time resolution [15,16], can be complex to operate at scale. Together, these constraints keep GSIS largely as a late-stage validation assay rather than an early, iterative design readout for differentiation and maturation protocols [11,17].

To address these limitations, several IOC platforms incorporate on-chip sensing to provide continuous insulin measurements, including electrophoretic immunoassays [18,19,20,21,22,23,24,25,26] and optical immunoassays such as fluorescence anisotropy [27,28,29,30]. Nevertheless, these approaches typically require added reagents and on-chip mixing, while the resulting dead volume and transport through channels can blur rapid secretion dynamics. Moreover, many implementations pool multiple islets to increase signal, obscuring heterogeneity. Microscopy-compatible IOC platforms have enabled single-islet measurements of upstream functional markers such as metabolism [31] and respiration dynamics [32], however, direct, real-time visualization of insulin secretion dynamics from individual islets remains a key gap. A sensor capable of fast, non-destructive functional readouts from single iPSC-derived islets would allow earlier evaluation of differentiation conditions, making it possible to iteratively refine differentiation protocols and produce islets with consistent glucose responsiveness. Such a sensor could also be used to pre-screen individual islets prior to transplantation.

## 2. Results

### 2.1. Microfluidic Platform for Single-Islet Microscopy

**Figure 1:**
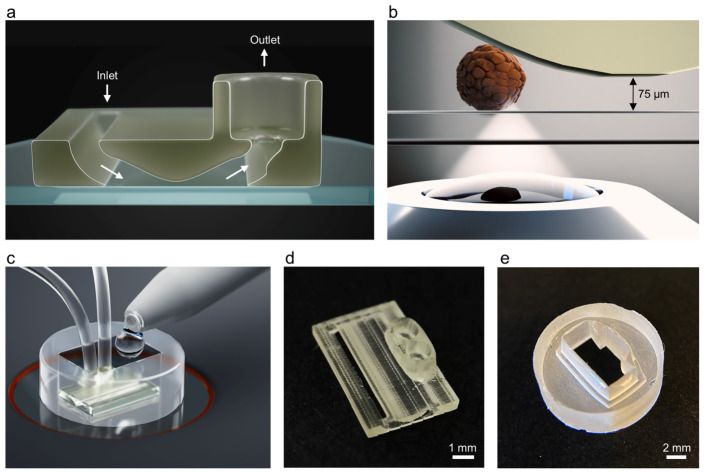
(**a**) Rendering of the 3D microfluidic design on a glass coverslip base. (**b**) Schematic of an islet trapped at the channel constriction and stabilized for imaging under continuous perfusion. (**c**) Rendering of the IOC mounted on a microscope stage with a PDMS spacer for pipette-based loading and reduced circulating volume. (**d**) Photograph of the two-photon polymerization-fabricated IOC. (**e**) Photograph of the PDMS spacer.

We have developed a microscopy-compatible islet-on-a-chip (IOC) platform that stabilizes individual functional human islets above a glass coverslip under continuous perfusion, enabling high-resolution optical readouts and quick stimulus switching. The device was fabricated using two-photon polymerization, which allows the creation of 3D biocompatible microfluidic devices (Supplementary Figure S1). A standard glass coverslip forms the chamber base, providing direct optical access for high-resolution microscopy (Figure 1a,b). Islets and media enter through a 1 mm wide inlet slit spanning the chip width, and the internal channel narrows from 1 mm to a 75 µm constriction near the base before expanding towards two outlet ports coupled to microfluidic tubing (500 µm ID, 1.5 mm OD) to limit downstream dead volume. The overall chip dimensions are 9 × 4.6 × 1 mm. Negative pressure applied at the outlets using stepper-motorized dual syringe pumps pulls islets to the constriction, where they are trapped and mechanically stabilized for imaging while media flows around them. To minimize mechanical stress on the islets, we operated at low perfusion rates. Finally, to reduce the circulating volume, support rapid stimulus exchange, and correctly position the IOC on the coverslip, we fabricated a custom PDMS spacer by casting PDMS in a 3D-printed mold. The spacer forms a compact loading chamber compatible with pipette-based loading (Figure 1c). Photographs of the microscopy-compatible IOC and PDMS spacer are shown in Figure 1d and Figure 1e, respectively.

### 2.2. Label-Free Optical Detection of Insulin Granule-Rich β-Cells

**Figure 2:**
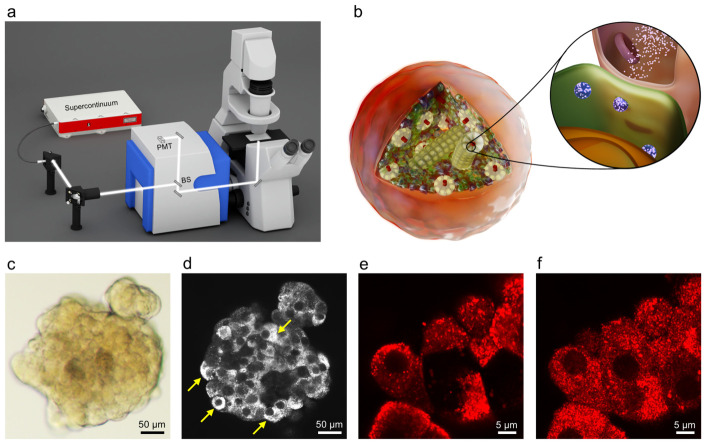
(**a**) Schematic of the BBCM optical setup, showing the supercontinuum light source, beamsplitter (BS), and photomultiplier tube (PMT). (**b**) Pancreatic islet with β-cells (green), α-cells, δ-cells, and blood vessels. When blood glucose levels rise, insulin granules fuse with the β-cell plasma membrane, allowing insulin to be released. (**c**) Brightfield image of a human pancreatic islet. (**d**) BBCM optical section showing heterogeneous cytoplasmic backscatter, with yellow arrows indicating cells exhibiting strong cytoplasmic backscatter. (**e**) Insulin immunofluorescence image identifying insulin-positive β-cells. (**f**) Corresponding BBCM image of the same region (displayed with a red lookup table), showing spatial correspondence between insulin-positive cells and high cytoplasmic backscatter.

We paired the microscopy-compatible IOC with broadband backscattering confocal microscopy (BBCM), a label-free optical sensing approach that has been described in detail elsewhere [33]. Briefly, BBCM uses a white light supercontinuum source and detects backscattered light in a confocal geometry (Figure 2a). By integrating the signal over a broad spectral range, BBCM averages wavelength-dependent intensity variations that can complicate monochromatic backscattering measurements, significantly improving contrast for sub-micron intracellular scatterers [33]. Additional optical and acquisition details are provided in Section 4.

BBCM is well suited for monitoring insulin secretion given that insulin is synthesized and stored in dense-core secretory granules within pancreatic β-cells [34]. When glucose levels rise, these granules fuse with the cell membrane to release insulin into the bloodstream (Figure 2b, inset). While the β-cells typically provide approximately 60% of the total islet mass [35], islets also contain other endocrine cell types with secretory granules (e.g., α-cells), and additional intracellular scatterers such as lipid droplets. However, given the distinctive crystalline core of insulin granules [34], combined with their abundance in β-cells [36], we expect insulin granule-rich β-cells to dominate the backscatter signal.

To test whether the IOC-BBCM platform could indeed visualize insulin granule-rich β-cells, we imaged functional human cadaveric islets with BBCM. A representative brightfield image is shown in Figure 2c. A BBCM optical section (Figure 2d) reveals strong cytoplasmic backscatter in a subset of cells, consistent with granule-rich endocrine cells (arrows). To validate that these high-contrast cells are β-cells, we performed insulin immunofluorescence staining on matched regions. Insulin-positive cells are readily identified by fluorescence (Figure 2e), and the corresponding BBCM image shows a strong spatial correspondence between insulin-positive cells and high cytoplasmic backscatter (Figure 2f). Together, these results demonstrate that BBCM can detect insulin granule-rich β-cells in intact human islets without exogenous labels.

### 2.3. IOC-BBCM Sensing of GSIS Dynamics in Single Islets

**Figure 3:**
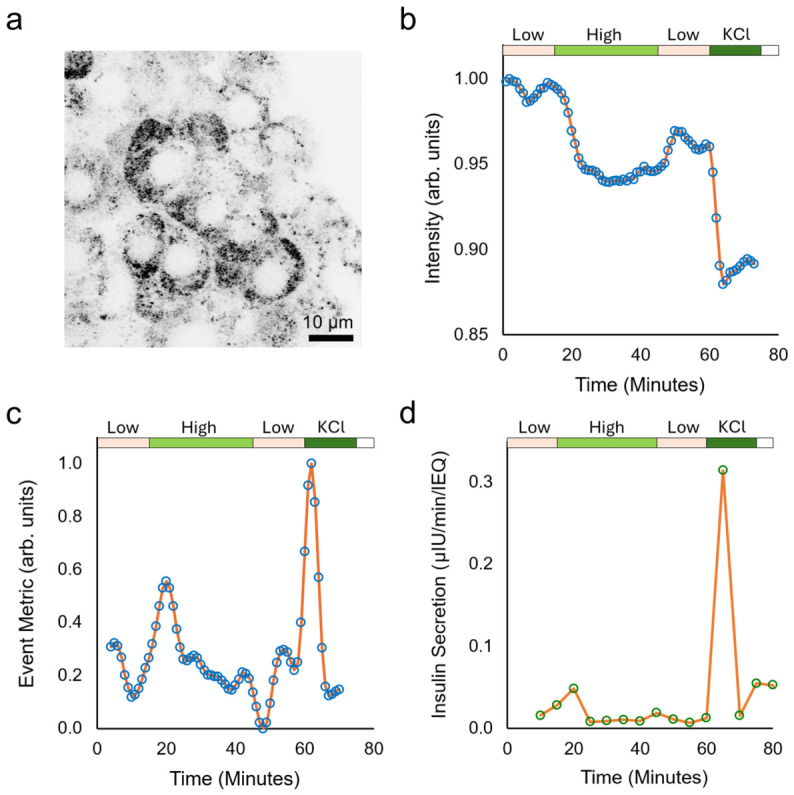
(**a**) First BBCM image from the single islet GSIS time series (inverted grayscale). (**b**) Background-normalized mean intensity trace derived from stabilized BBCM frames of the selected single islet, with stimulus timing indicated. (**c**) Event metric (windowed step detector) computed from the normalized trace, highlighting abrupt decreases at high-glucose and KCl transitions. (**d**) Insulin concentration in effluent collected every 5 min from the 10-islet population loaded in the IOC and quantified by ELISA, showing increased secretion during high glucose and KCl stimulation.

We next employed the IOC-BBCM platform to monitor glucose-stimulated insulin secretion (GSIS) dynamics in individual human islets. Ten islets (100–150 µm diameter) were loaded into the IOC in Krebs–Ringer bicarbonate (KRB) buffer containing 2.8 mM glucose and equilibrated for 45 min under perfusion. The ten islets provided sufficient insulin signal for downstream ELISA analysis of the collected effluent. However, the BBCM data was acquired from one selected islet and was not averaged across the loaded islet population. Because the IOC is optically transparent, the loaded islets could be monitored in transmission mode through the microscope eyepiece, allowing trapping and positioning of the islets to be easily verified before BBCM imaging. After equilibration, the islets were sequentially stimulated with 2.8 mM glucose for 15 min, 20 mM glucose for 30 min, 2.8 mM glucose for 15 min, and 30 mM KCl for 15 min. BBCM images of the selected islet were acquired once per minute throughout the protocol, while effluent from the full 10-islet population was collected every 5 min for insulin quantification by ELISA. The first image from the single-islet BBCM time series is shown in Figure 3a.

To extract a robust, label-free optical readout of stimulus-linked granule dynamics from the BBCM time series, we first stabilized the sequence using translation-only registration to suppress perfusion-induced motion. Inter-frame shifts were estimated sequentially, and the cumulative transform was applied to align all frames to the first frame. We then computed a simple intensity-based sensor metric, which was defined as the mean intensity of each stabilized BBCM frame, normalized by the mean intensity within a user-defined background region-of-interest. This per-frame normalization reduces sensitivity to slow fluctuations in illumination or detector gain. Because strong BBCM contrast spatially corresponded with insulin-positive β-cells, and given that insulin secretory granules are abundant, dense intracellular scatterers, we interpret decreases in this metric as reflecting stimulus-linked changes in granule-associated backscatter within the imaged islet. The normalized BBCM signal (Figure 3b) showed step-like decreases at the onset of high glucose and following KCl depolarization. While we believe these changes are dominated by alterations in granule-associated backscatter, other secretion-associated changes in cell morphology, intracellular refractive index, or organelle organization may also contribute to the measured signal. To highlight the step-like transitions, we applied a windowed step detector that reports the magnitude of abrupt decreases in the normalized BBCM trace (Figure 3c). The major events aligned well with increased insulin in downstream effluent from the 10-islet population as measured by ELISA (Figure 3d).

## 3. Conclusions

We have demonstrated a microscopy-compatible islet-on-a-chip (IOC) integrated with broadband backscattering confocal microscopy (BBCM) that enables label-free, non-destructive monitoring of insulin granules in functional human islets under controlled perfusion. Using translation-only motion correction and per-frame background normalization, the platform provides a simple optical readout that reports stimulus-linked transitions during glucose stimulation and KCl depolarization, with these transitions agreeing well with ELISA measurements on downstream effluent.

A key advantage of IOC-BBCM sensor is its microscopy compatibility and therefore, by extension, its modularity. The same trapped islet can be interrogated by additional optical modalities without altering the microfluidic workflow. For example, two-photon NAD(P)H imaging could provide a complementary label-free metabolic readout [31]. There is also the potential for integrating biochemical-sensitive Raman microscopy and chromatin-sensitive confocal light absorption and scattering spectroscopic (CLASS) microscopy. Both of these modalities have previously been demonstrated as reliable label-free methods for assessing the differentiation trajectories of stem-cell derived organoids [37].

Thus, as islet replacement therapies advance, there is a critical need for non-destructive methods that combine direct functional readouts with high-content, single-islet measurements. The IOC-BBCM sensor shown here provides a label-free method for monitoring GSIS-linked dynamics in individual functional islets. This sensor could potentially allow GSIS measurements to be performed earlier in the differentiation protocol, accelerating protocol optimization and early identification of failed differentiation trajectories. More broadly, the IOC-BBCM sensor could support the development of iPSC-derived islets with insulin secretion properties closer to those of native human islets, a goal that remains difficult to achieve with existing methods.

## 4. Materials & Methods

Two-photon polymerization: The IOC CAD design, generated in SolidWorks 2018, was exported to a stereolithography file format, with settings optimized to achieve fine polygon tessellation. Settings for laser-writing were then defined in DeScribe 2.5.5. The chip was fabricated with two-photon polymerization technology (Nanoscribe Photonic Professional GT2, Nanoscribe GmbH, Eggenstein-Leopoldshafen, Germany) using IP-Q photoresist, developed in PGMEA solution (484431, Sigma-Aldrich, St. Louis, MO, USA) for 20 min, and then rinsed with isopropanol for 5 min.

PDMS spacer: The mold was designed in SolidWorks and printed using a Form 2 printer (Formlabs, Somerville, MA, USA). The mold was then placed in deionized water and left for 1 h. It was then cured in an oven for 24 h at 65 °C and washed with deionized water. Following these steps, PDMS was placed in the mold and left to set at room temperature.

Broadband backscattering confocal microscopy: A Zeiss LSM 510 META laser scanning confocal microscope frame, which is also capable of fluorescence imaging, was used. The BBCM system utilizes a broadband coherent supercontinuum source (WhiteLase-Micro Compact Supercontinuum, NKT Photonics, Birkerød, Denmark) that consists of a Ti–sapphire pump laser coupled to a photonic crystal fiber. The collimated white light beam (425–800 nm) from the supercontinuum source is aligned with free-space coupling optics at the microscope entrance port. A high NA = 1.4 achromatic microscope objective (Zeiss Plan-Apochromat 63×, Jena, Germany) with low chromatic aberration was used to focus the beam. The backscattered light passes through a collection pinhole, which blocks most of the light originating above and below the focal plane. This allows only the light scattered within the focal volume to be delivered to the PMT for detection. The x, y, and z full width half maximum dimensions of the white light point spread function are 256 nm, 262 nm, and 1043 nm, respectively. The pixel dwell time for all measurements was 1.6 μs, allowing 512 × 512 pixel images to be acquired in 983 ms. For the GSIS time-lapse experiment, images were acquired once per minute throughout the stimulation protocol. The full field of view for the chosen objective was 142.86 × 142.86 µm.

Human pancreatic islets: Cadaveric human islets were obtained from Prodo Laboratories (Aliso Viejo, CA, USA) and maintained according to the protocol provided by the company. The research carried out complied with all relevant ethical regulations, as provided by Beth Israel Deaconess Medical Center. For initial inspection, a subset of the islets were fixed in 4% paraformaldehyde overnight at 4 °C and embedded in 4% low-melting-point agarose in PBS for sectioning. Tissue sectioning, along with hematoxylin and eosin (H&E) staining, was performed by the histology core facility at Beth Israel Deaconess Medical Center. Representative images are shown in Supplementary Figure S2.

Immunohistochemistry: Islets were fixed in 4% PFA solution for 14 h. After fixation, islets were washed in PBS (15 min, 3 times) at room temperature and placed in permeabilization solution (1% Triton-X 100, 2% BSA and PBS) for 45 min. Islets were then blocked in 5% BSA solution for 1.5 h and washed 3 times (15 min each time) with washing solution (0.1% Tween 20, 2% BSA and PBS). We used a recombinant anti-insulin antibody (ab181547, 1:600 dilution, Abcam, Cambridge, UK) for the insulin staining. Alexa Fluor 488 (Goat Anti-Rabbit, a11034, 1:400 dilution, Invitrogen, Carlsbad, CA, USA) was used as the secondary antibody. Islets were incubated with the primary antibody for 14 h at 4 °C and washed 3 times (15 min each time) using washing solution (0.1% Tween 20, 2% BSA and PBS) the next day at room temperature. After the washing step, islets were incubated with the secondary antibody for 2 h. The last step before imaging was a washing step using washing solution (0.1% Tween 20, 2% BSA and PBS) for 15 min and PBS for 15 min (2 times).

Insulin secretion assay: Stimulation media was added to the IOC approximately every 5 min during the stimulation protocol using a pipette. Collected perfusate (15 µL/min) was analyzed for insulin content using an ELISA-based detection kit (80-INSHUU-E01.1, ALPCO Diagnostics, Salem, NH, USA) and a microplate reader operating at a 450 nm absorbance wavelength (SpectraMax i3x, Molecular Devices, San Jose, CA, USA).

Image stabilization: To suppress perfusion-induced lateral motion during time-lapse BBCM imaging, each frame in the GSIS time series was motion-corrected using translation-only image registration in MATLAB R2022b (MathWorks, Natick, MA, USA). Raw image stacks were imported in their native Zeiss LSM format, and the first frame was used to define a fixed reference coordinate system, serving as the global reference for alignment. For each subsequent frame, the lateral translation mapping the current frame to the immediately preceding raw frame was estimated using correlation-based registration. These stepwise translations were cumulatively composed to obtain a net transform mapping each frame into the coordinate system of the first frame. Each frame was then resampled into the reference geometry using the cumulative transform with an output view fixed to the first frame. Pixels mapped outside the field of view were assigned a value of zero. Stabilized frames were cropped in a subsequent step to retain only the region containing valid image content.

Background-normalized optical readout and event detection: To derive an intensity-based optical readout from stabilized BBCM image sequences, pre-stabilized and cropped TIFF frames were analyzed in MATLAB. A background region of interest (ROI) was selected manually on the first frame using a freehand mask in an area free of islet signal. The same background mask was applied to all frames. For each frame, the mean intensity of all pixels in the image and the mean intensity within the background ROI were computed. A dimensionless, per-frame normalized intensity metric was then calculated.

To highlight abrupt, stimulus-linked transitions in the normalized trace, an event metric was computed using a windowed step detector. The normalized trace was smoothed with a 3-frame moving average. For each time point, the mean of the preceding 3 frames and the mean of the subsequent 3 frames were computed, and the event metric was defined as the negative of the difference between these window means. This meant step-like decreases in the BBCM signal provided positive event amplitudes. For visualization, the event metric was offset by the absolute value of the minimum value and normalized to the maximum value.

## Data Availability

The original data presented in the study are openly available in Figshare at https://doi.org/10.6084/m9.figshare.31758442. MATLAB code for generating the background-normalized optical readout and event metric is available via GitHub at https://github.com/mfcoughl/IOC-BBCM (accessed on 30 April 2026).

## Supplementary Materials

The following supporting information can be downloaded at: https://www.mdpi.com/article/10.3390/s26103069/s1, Figure S1: Fabrication of the IOC using two-photon polymerization. The simultaneous absorption of photons within the elliptical focus of a laser beam leads to polymerization of the photoresist (inset), with three-dimensional scanning of the laser beam through the photoresist allowing complex nanostructures to be easily created; Figure S2: H&E staining of representative pancreatic islets. Brightfield micrographs of hematoxylin and eosin (H&E)-stained sections from a subset of islets. The images highlight expected islet morphology and cellular architecture.
