# Supplementary material for: Label-Free Optical Sensor for Real-Time Monitoring of Insulin Secretion from Single Human Pancreatic Islets
Source: Sensors (Basel). 2026 May 13;26(10):3069. doi: 10.3390/s26103069 (PMC13211296; doi:10.3390/s26103069)
Supplement: Supplementary file 1 [file sensors-26-03069-s001.zip › sensors-4269626-supplementary.pdf]

Label-free optical sensor for real-time monitoring of insulin secretion from single human pancreatic islets

Mark F. Coughlan*, Lei Zhang, Umar Khan, Xuejun Zhang, Paul K. Upputuri, Maria Glyavina, Yuri N. Zakharov, Le Qiu & Lev T. Perelman*

Center for Advanced Biomedical Imaging and Photonics
Beth Israel Deaconess Medical Center
Harvard University

SUPPLEMENTARY INFORMATION

* Correspondence: mfcoughl@bidmc.harvard.edu; ltperel@bidmc.harvard.edu

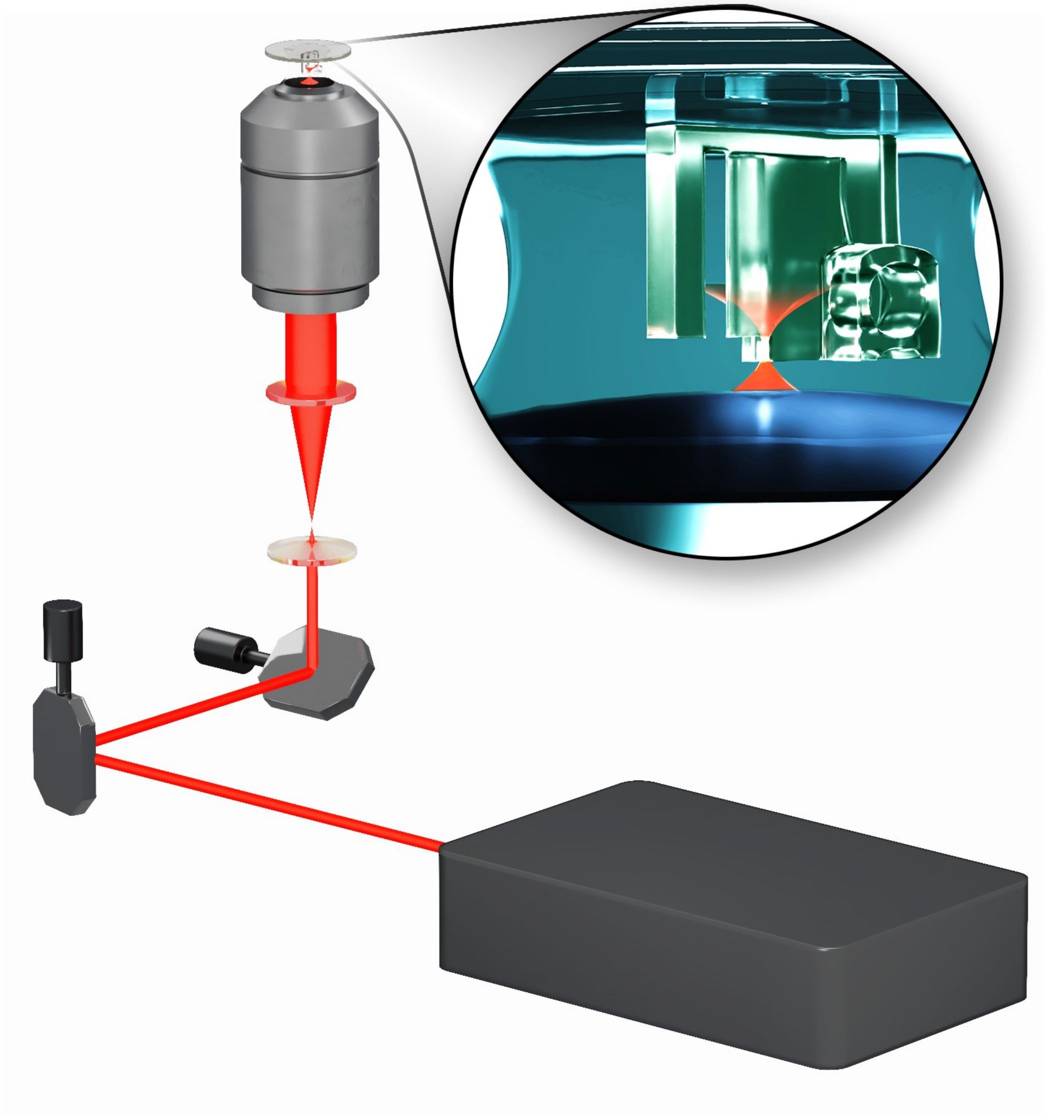

Supplementary Figure S1. Fabrication of the IOC using two-photon polymerization. The simultaneous absorption of photons within the elliptical focus of a laser beam leads to polymerization of the photoresist (inset), with three-dimensional scanning of the laser beam through the photoresist allowing complex nanostructures to be easily created.

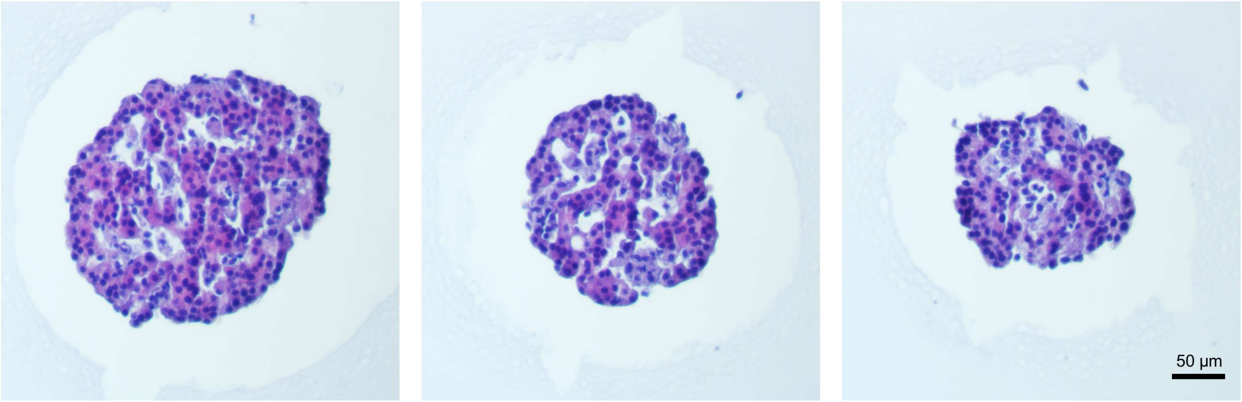

Supplementary Figure S2. H&E staining of representative pancreatic islets. Brightfield micrographs of hematoxylin and eosin (H&E)-stained sections from a subset of islets. The images highlight expected islet morphology and cellular architecture.
